# *N*-[5-Methyl-2-(2-nitro­phen­yl)-4-oxo-1,3-thia­zolidin-3-yl]pyridine-3-carboxamide monohydrate

**DOI:** 10.1107/S1600536811000481

**Published:** 2011-01-08

**Authors:** Mehmet Akkurt, Ísmail Çelik, Hale Demir, Sumru Özkırımlı, Orhan Büyükgüngör

**Affiliations:** a Department of Physics, Faculty of Sciences, Erciyes University, 38039 Kayseri, Turkey; b Department of Physics, Faculty of Arts and Sciences, Cumhuriyet University, 58140 Sivas, Turkey; c Department of Pharmaceutical Chemistry, Faculty of Pharmacy, Istanbul University, 34116 Beyazit, Istanbul, Turkey; d Department of Physics, Faculty of Arts and Sciences, Ondokuz Mayıs University, 55139 Samsun, Turkey

## Abstract

In the title compound, C_16_H_14_N_4_O_4_S·H_2_O, the benzene and pyridine rings make a dihedral angle of 85.8 (1)°. Both enanti­omers of the chiral title compound are statistically disordered over the same position in the unit cell. The methyl and carbonyl group attached to the stereogenic center (C_5_ of the thia­zolidine ring) were therefore refined with common site-occupation factors of 0.531 (9) and 0.469 (9), respectively, for each stereoisomer. In the crystal, inter­molecular N—H⋯O, O—H⋯O and O—H⋯N hydrogen bonds link the mol­ecules, forming a three-dimensional supra­molecular network. The crystal structure further shows π–π stacking inter­actions [centroid–centroid distance = 3.5063 (13) Å] between the pyridine rings.

## Related literature

For the biological and pharmacological properties of pyridine-3-carboxamide derivatives, see: Balzarini *et al.* (2009 ▶); Baumbach *et al.* (1995 ▶); Girgis *et al.* (2006 ▶); Guzel & Salman (2009 ▶); Kuramochi *et al.* (2005 ▶); Moëll *et al.* (2009 ▶); Slominska *et al.* (2008 ▶); Ur *et al.* (2004 ▶); Vigorita *et al.* (1992 ▶). For bond-length data, see: Allen *et al.* (1987 ▶).
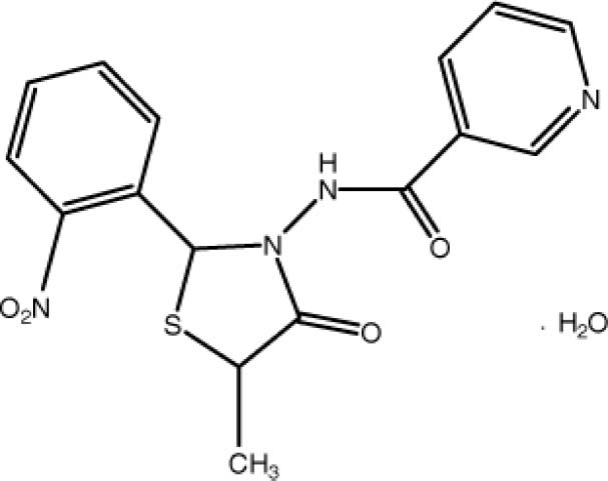


## Experimental

### 

#### Crystal data

C_16_H_14_N_4_O_4_S·H_2_O
*M* *_r_* = 376.40
Triclinic, 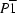

*a* = 8.1399 (4) Å
*b* = 8.4106 (4) Å
*c* = 15.0274 (7) Å
α = 92.957 (4)°
β = 104.176 (4)°
γ = 116.792 (4)°
*V* = 874.66 (8) Å^3^
*Z* = 2
Mo *K*α radiation
μ = 0.22 mm^−1^
*T* = 296 K
0.62 × 0.55 × 0.49 mm

#### Data collection

Stoe IPDS 2 diffractometer
Absorption correction: integration (*X-RED32*; Stoe & Cie, 2002 ▶) *T*_min_ = 0.875, *T*_max_ = 0.899
11714 measured reflections
3963 independent reflections
3192 reflections with *I* > 2σ(*I*)
*R*_int_ = 0.038

#### Refinement

*R*[*F* ^2^ > 2σ(*F* ^2^)] = 0.043
*wR*(*F* ^2^) = 0.121
*S* = 1.04
3963 reflections
285 parameters
15 restraints
H atoms treated by a mixture of independent and constrained refinement
Δρ_max_ = 0.27 e Å^−3^
Δρ_min_ = −0.27 e Å^−3^

### 

Data collection: *X-AREA* (Stoe & Cie, 2002 ▶); cell refinement: *X-AREA*; data reduction: *X-RED32* (Stoe & Cie, 2002 ▶); program(s) used to solve structure: *SIR97* (Altomare *et al.*, 1999 ▶); program(s) used to refine structure: *SHELXL97* (Sheldrick, 2008 ▶); molecular graphics: *ORTEP-3* (Farrugia, 1997 ▶); software used to prepare material for publication: *WinGX* (Farrugia, 1999 ▶).

## Supplementary Material

Crystal structure: contains datablocks global, I. DOI: 10.1107/S1600536811000481/im2255sup1.cif

Structure factors: contains datablocks I. DOI: 10.1107/S1600536811000481/im2255Isup2.hkl

Additional supplementary materials:  crystallographic information; 3D view; checkCIF report

## Figures and Tables

**Table 1:** Hydrogen-bond geometry (Å, °)

*D*—H⋯*A*	*D*—H	H⋯*A*	*D*⋯*A*	*D*—H⋯*A*
N3—H*N*1⋯O*W*1^i^	0.86 (2)	1.96 (2)	2.804 (2)	167 (2)
O*W*1—H*W*1⋯O3*A*^ii^	0.81 (2)	2.02 (2)	2.806 (1)	163 (4)
O*W*1—H*W*2⋯N4^iii^	0.80 (2)	2.01 (2)	2.803 (2)	173 (3)

Symmetry codes: (i) 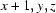; (ii) 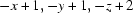; (iii) 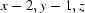.

**Fig. 1.**
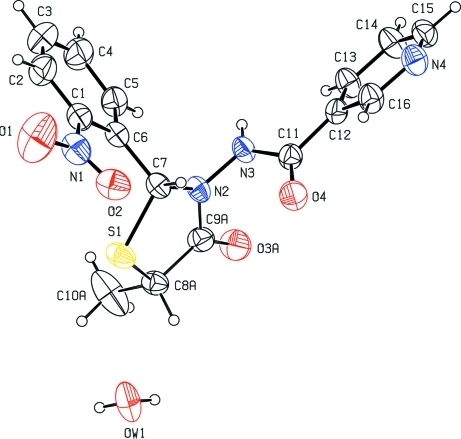
View of the title molecule with the atom numbering scheme. Displacement ellipsoids for non-H atoms are drawn at the 30% probability level. Only the S-enantiomer is shown.

**Fig. 2.**
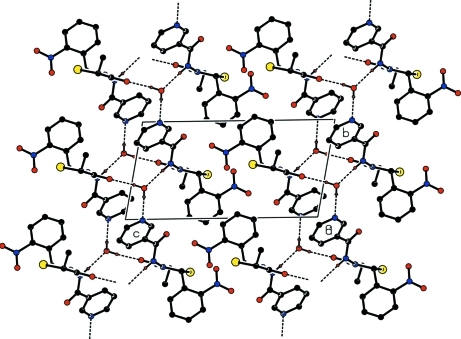
Packing diagram of (I) viewed down the a-axis and showing the hydrogen bonding pattern. All hydrogen atoms not involved in hydrogen bonding have been omitted for clarity. Only the S-enantiomers are shown.

## supplementary crystallographic information

### Comment

Pyridine-3-carboxamide derivatives have gained attention because of their cytoprotective (Slominska *et al.*, 2008), sodium-calcium exchanger (NCX) inhibitory (Kuramochi *et al.*, 2005), vasodilatory (Baumbach *et al.*, 1995), and cytotoxic (Girgis *et al.*, 2006) properties. An inhibitory effect of pyridine-3-carboxamide on enterovirus replication and chemokine secretion has also been recently reported (Moëll *et al.*, 2009). Here, we combine the pyridine-3-carboxamide moiety with a thiazolidinone moiety, which has shown antimycobacterial (Guzel *et al.*, 2009), antimicrobial (Ur *et al.*, 2004), anticancer (Vigorita *et al.*, 1992) and antiviral (Balzarini *et al.*, 2009) activities. Design and synthesis of bioactive molecules bearing both 4-thiazolidinone and pyridine-3-carboxamide groups take advantage of the diverse biological activities of the two scaffolds.

In the title compound (I), (Fig. 1), the bond lengths and bond angles are within normal range (Allen *et al.*, 1987). The benzene (C1–C6) and pyridine (N4/C12–C16) rings in (I) make a dihedral angle of 85.8 (1)° with each other.

Both enantiomers of the chiral title compound are statistically disordered over the same position in the unit cell. The methyl and carbonyl group attached to the stereogenic center (C_(5)_ of the thiazolidine ring) were therefore refined with common site occupation factors of 0.531 (9) and 0.469 (9), respectively, for each stereoisomer. Atoms C8A and C8B show strange thermal parameters due to the observed disorder.

The molecular packing (Fig. 2), is stabilized by the intermolecular N—H···O, O—H···O and O—H···N hydrogen bonds connecting the molecules to form a three-dimensional supramolecular network (Table 1). Additionally, a π-π stacking interaction in the structure was observed between the pyridine rings of the two adjacent molecules [*Cg*3···*Cg*3*^iv^* = 3.5063 (13) Å, symmetry code (*iv*) 3 - *x*, 2 - *y*, 2 - *z*; *Cg*3 is a centroid of the pyridine ring (N4/C12—C16)].

### Experimental

0.01 mol of *N*'-(2-nitrobenzylidine)pyridine-3-carbohydrazide was reacted with 0.028 mol of 2-mercaptopropanoic acid in anhydrous benzene for 18 h using a Dean-Stark trap. Excess benzene was removed under reduced pressure. The residue was triturated with saturated sodium bicarbonate solution. The separated solid was filtered, washed with water and crystallized from methanol to obtain a white crystalline solid. Yield: 70.11%; m.p.: 378.2–382.1 K. UV (EtOH) λ max: 212.2, 220.0, 255.4 nm. IR (KBr) υ: 1681 (amide C=O), 1707 (thia C=O) cm^-1^; ^1^H-NMR (DMSO-d_6_, 500 MHz): 1.48, 1.52 (3*H*, 2 d, J=7.3 Hz, 6.8 Hz, CH_3_-thia.), 4.10 (1*H*, q, J= 7.0 Hz, H5-thia.), 4.21 (1*H*, dq, J= 6.8, 1.96 Hz, H5-thia.), 6.22 (1*H*, d, J=2 Hz, H2-thia), 7.51 (1*H*, dd, J=4.8 Hz, 4.4 Hz, H5-pyridine), 7.61–7.65 (1*H*, m, 2-C_6_H_4_—H4-thia.), 7.85–7.90 (2*H*, m, 2-C_6_H_4_-(H5,6)-thia.), 8.04–8.11 (2*H*, m, 2-C_6_H_4_—H3-thia. ve H4-pyridine), 8.73 (1*H*, dd, J=8.3 Hz, 2.0 Hz, H6-pyridine), 8.71, 8.79 (1*H*, 2 d, J=2.4 Hz, 2.4 Hz, H2-pyridine), 11.04, 11.05 (1*H*, 2 s, CONH) p.p.m.; ESI– (m/*z*, relative abundance): 358.13 ([M—H+1]^-^, 17.77), 357.13 ([M—H]^-^, 100). Analysis calculated for C_16_H_14_N_4_O_4_S.H_2_O: C 51.06, H 4.28, N 14.89%. Found: C 51.21, H 3.73, N 14.83%.

### Refinement

H atoms of the water molecule were found from a difference Fourier map and were refined with distance restraints of O–H = 0.82 Å, H···H = 1.23 Å, and with *U*_iso_(H) = 1.5 *U*_eq_(O). The N-bound H atom was located from the Fourier synthesis and was refined with a distance restraint of N–H = 0.86 Å, and with *U*_iso_(H) = 1.2 *U*_eq_(N). C-bound H atoms were placed geometrically (C—H = 0.93–0.98 Å) and refined as riding with *U*_iso_(H) = 1.2 or 1.5*U*_eq_(C).

### Crystal data

**Table:**

C_16_H_14_N_4_O_4_S·H_2_O	*Z* = 2
*M**_r_* = 376.40	*F*(000) = 392
Triclinic, *P*1	*D*_x_ = 1.429 Mg m^−^^3^
Hall symbol: -P 1	Mo *K*α radiation, λ = 0.71073 Å
*a* = 8.1399 (4) Å	Cell parameters from 15771 reflections
*b* = 8.4106 (4) Å	θ = 2.8–28.0°
*c* = 15.0274 (7) Å	µ = 0.22 mm^−^^1^
α = 92.957 (4)°	*T* = 296 K
β = 104.176 (4)°	Block, colourless
γ = 116.792 (4)°	0.62 × 0.55 × 0.49 mm
*V* = 874.66 (8) Å^3^	

### Data collection

**Table:**

Stoe IPDS 2 diffractometer	3963 independent reflections
Radiation source: sealed X-ray tube, 12 x 0.4 mm long-fine focus	3192 reflections with *I* > 2σ(*I*)
plane graphite	*R*_int_ = 0.038
Detector resolution: 6.67 pixels mm^-1^	θ_max_ = 27.5°, θ_min_ = 2.8°
ω scans	*h* = −10→10
Absorption correction: integration (*X-RED32*; Stoe & Cie, 2002)	*k* = −10→10
*T*_min_ = 0.875, *T*_max_ = 0.899	*l* = −19→18
11714 measured reflections	

### Refinement

**Table:**

Refinement on *F*^2^	Primary atom site location: structure-invariant direct methods
Least-squares matrix: full	Secondary atom site location: difference Fourier map
*R*[*F*^2^ > 2σ(*F*^2^)] = 0.043	Hydrogen site location: inferred from neighbouring sites
*wR*(*F*^2^) = 0.121	H atoms treated by a mixture of independent and constrained refinement
*S* = 1.04	*w* = 1/[σ^2^(*F*_o_^2^) + (0.057*P*)^2^ + 0.1358*P*] where *P* = (*F*_o_^2^ + 2*F*_c_^2^)/3
3963 reflections	(Δ/σ)_max_ = 0.001
285 parameters	Δρ_max_ = 0.27 e Å^−^^3^
15 restraints	Δρ_min_ = −0.27 e Å^−^^3^

### Special details

**Table:**

Geometry. Bond distances, angles *etc*. have been calculated using the rounded fractional coordinates. All su's are estimated from the variances of the (full) variance-covariance matrix. The cell e.s.d.'s are taken into account in the estimation of distances, angles and torsion angles
Refinement. Refinement on *F*^2^ for ALL reflections except those flagged by the user for potential systematic errors. Weighted *R*-factors *wR* and all goodnesses of fit *S* are based on *F*^2^, conventional *R*-factors *R* are based on *F*, with *F* set to zero for negative *F*^2^. The observed criterion of *F*^2^ > σ(*F*^2^) is used only for calculating -*R*-factor-obs *etc*. and is not relevant to the choice of reflections for refinement. *R*-factors based on *F*^2^ are statistically about twice as large as those based on *F*, and *R*-factors based on ALL data will be even larger.

### Fractional atomic coordinates and isotropic or equivalent isotropic displacement parameters (Å^2^)

**Table:**

	*x*	*y*	*z*	*U*_iso_*/*U*_eq_	Occ. (<1)
S1	0.56133 (7)	0.45061 (8)	0.61403 (3)	0.0699 (2)	
O1	0.7192 (5)	0.2528 (4)	0.38302 (13)	0.1562 (12)	
O2	0.7900 (2)	0.4795 (3)	0.48092 (11)	0.0856 (6)	
O3A	0.7493 (19)	0.5809 (14)	0.8800 (8)	0.091 (3)	0.531 (9)
O4	1.1251 (2)	0.84381 (17)	0.77262 (11)	0.0811 (5)	
N1	0.7598 (3)	0.3251 (3)	0.46237 (12)	0.0771 (6)	
N2	0.84041 (19)	0.50765 (19)	0.76035 (9)	0.0551 (4)	
N3	1.0289 (2)	0.56998 (19)	0.81531 (10)	0.0563 (4)	
N4	1.6987 (2)	0.9892 (2)	0.90983 (13)	0.0741 (6)	
C1	0.7803 (3)	0.2246 (3)	0.53763 (13)	0.0645 (6)	
C2	0.7799 (4)	0.0637 (3)	0.5129 (2)	0.0927 (9)	
C3	0.7969 (4)	−0.0385 (4)	0.5785 (3)	0.1087 (13)	
C4	0.8097 (4)	0.0165 (3)	0.6687 (2)	0.0947 (10)	
C5	0.8058 (3)	0.1748 (3)	0.69268 (15)	0.0707 (7)	
C6	0.7942 (2)	0.2861 (2)	0.62885 (12)	0.0539 (5)	
C7	0.7962 (2)	0.4616 (2)	0.65973 (11)	0.0527 (5)	
C8A	0.5297 (5)	0.4937 (9)	0.7236 (2)	0.0587 (13)	0.531 (9)
C9A	0.7153 (13)	0.5370 (11)	0.7958 (6)	0.0610 (19)	0.531 (9)
C10A	0.3540 (12)	0.3317 (16)	0.7341 (6)	0.126 (4)	0.531 (9)
C11	1.1625 (3)	0.7433 (2)	0.81826 (12)	0.0567 (5)	
C12	1.3603 (2)	0.7956 (2)	0.87999 (12)	0.0529 (5)	
C13	1.3975 (3)	0.7257 (3)	0.95825 (13)	0.0648 (6)	
C14	1.5852 (3)	0.7867 (3)	1.01129 (15)	0.0738 (7)	
C15	1.7293 (3)	0.9187 (3)	0.98500 (15)	0.0681 (6)	
C16	1.5163 (3)	0.9272 (2)	0.85835 (14)	0.0679 (6)	
C8B	0.5041 (6)	0.3820 (10)	0.7244 (3)	0.0592 (16)	0.469 (9)
C9B	0.7003 (12)	0.4774 (11)	0.7995 (6)	0.0513 (16)	0.469 (9)
C10B	0.3623 (15)	0.4314 (15)	0.7441 (7)	0.093 (3)	0.469 (9)
O3B	0.7218 (17)	0.5168 (14)	0.8810 (8)	0.076 (2)	0.469 (9)
OW1	0.0315 (2)	0.3195 (2)	0.93103 (12)	0.0931 (6)	
H4	0.82100	−0.05310	0.71360	0.1130*	
H7	0.89160	0.56040	0.63860	0.0630*	
H8A	0.50960	0.60000	0.72670	0.0710*	0.531 (9)
H5	0.81110	0.20840	0.75370	0.0850*	
H10B	0.33860	0.35480	0.79400	0.1890*	0.531 (9)
H10C	0.37100	0.22610	0.72940	0.1890*	0.531 (9)
H13	1.29640	0.63760	0.97530	0.0780*	
H14	1.61340	0.73910	1.06390	0.0890*	
H15	1.85600	0.96150	1.02190	0.0820*	
H16	1.49240	0.97470	0.80510	0.0810*	
H10A	0.24130	0.31160	0.68570	0.1890*	0.531 (9)
HN1	1.048 (3)	0.499 (2)	0.8508 (13)	0.062 (5)*	
H2	0.76800	0.02530	0.45140	0.1110*	
H3	0.79980	−0.14520	0.56220	0.1300*	
H8B	0.45250	0.25050	0.71860	0.0710*	0.469 (9)
H10D	0.24250	0.36760	0.69470	0.1400*	0.469 (9)
H10E	0.41040	0.55960	0.74800	0.1400*	0.469 (9)
H10F	0.34190	0.39900	0.80230	0.1400*	0.469 (9)
HW1	0.078 (4)	0.355 (4)	0.9872 (13)	0.120 (11)*	
HW2	−0.068 (3)	0.230 (3)	0.924 (2)	0.126 (11)*	

### Atomic displacement parameters (Å^2^)

**Table:**

	*U*^11^	*U*^22^	*U*^33^	*U*^12^	*U*^13^	*U*^23^
S1	0.0665 (3)	0.1068 (4)	0.0450 (2)	0.0532 (3)	0.0084 (2)	0.0109 (2)
O1	0.245 (3)	0.159 (2)	0.0532 (10)	0.088 (2)	0.0453 (15)	0.0094 (12)
O2	0.0975 (11)	0.1172 (13)	0.0581 (8)	0.0619 (10)	0.0255 (8)	0.0329 (9)
O3A	0.103 (6)	0.130 (7)	0.046 (3)	0.069 (5)	0.009 (3)	−0.007 (4)
O4	0.0813 (9)	0.0583 (7)	0.0893 (10)	0.0317 (7)	0.0032 (8)	0.0239 (7)
N1	0.0713 (10)	0.1071 (14)	0.0478 (9)	0.0377 (10)	0.0208 (8)	0.0090 (9)
N2	0.0463 (7)	0.0679 (8)	0.0414 (7)	0.0231 (6)	0.0062 (5)	0.0082 (6)
N3	0.0466 (7)	0.0540 (7)	0.0549 (8)	0.0182 (6)	0.0038 (6)	0.0145 (6)
N4	0.0560 (9)	0.0652 (9)	0.0838 (12)	0.0139 (7)	0.0225 (8)	0.0155 (8)
C1	0.0540 (9)	0.0745 (11)	0.0580 (10)	0.0266 (8)	0.0150 (8)	0.0051 (8)
C2	0.0887 (16)	0.0873 (15)	0.0903 (17)	0.0380 (13)	0.0218 (13)	−0.0130 (13)
C3	0.114 (2)	0.0742 (15)	0.131 (3)	0.0498 (15)	0.0206 (19)	−0.0047 (16)
C4	0.0990 (18)	0.0634 (12)	0.107 (2)	0.0358 (12)	0.0121 (15)	0.0204 (12)
C5	0.0716 (12)	0.0611 (10)	0.0665 (12)	0.0247 (9)	0.0125 (9)	0.0166 (9)
C6	0.0446 (8)	0.0596 (9)	0.0512 (9)	0.0209 (7)	0.0117 (7)	0.0111 (7)
C7	0.0508 (8)	0.0626 (9)	0.0425 (8)	0.0253 (7)	0.0131 (6)	0.0155 (7)
C8A	0.061 (2)	0.071 (3)	0.0513 (18)	0.038 (2)	0.0153 (15)	0.0157 (17)
C9A	0.070 (3)	0.069 (4)	0.051 (3)	0.041 (3)	0.014 (2)	0.012 (3)
C10A	0.066 (4)	0.190 (9)	0.077 (5)	0.021 (5)	0.019 (3)	0.056 (6)
C11	0.0582 (9)	0.0502 (8)	0.0557 (9)	0.0233 (7)	0.0124 (8)	0.0097 (7)
C12	0.0519 (8)	0.0440 (7)	0.0542 (9)	0.0174 (6)	0.0135 (7)	0.0069 (6)
C13	0.0504 (9)	0.0669 (10)	0.0607 (10)	0.0152 (8)	0.0138 (8)	0.0192 (8)
C14	0.0571 (10)	0.0843 (13)	0.0658 (11)	0.0242 (9)	0.0118 (9)	0.0250 (10)
C15	0.0485 (9)	0.0713 (11)	0.0739 (12)	0.0220 (8)	0.0151 (9)	0.0082 (9)
C16	0.0628 (11)	0.0543 (9)	0.0709 (12)	0.0160 (8)	0.0168 (9)	0.0182 (8)
C8B	0.051 (2)	0.071 (4)	0.053 (2)	0.027 (2)	0.0144 (16)	0.017 (2)
C9B	0.050 (2)	0.065 (4)	0.039 (2)	0.027 (3)	0.0129 (16)	0.018 (3)
C10B	0.080 (5)	0.150 (8)	0.073 (3)	0.071 (6)	0.031 (3)	0.021 (5)
O3B	0.060 (2)	0.113 (6)	0.043 (3)	0.031 (4)	0.0160 (18)	0.015 (4)
OW1	0.0800 (10)	0.0681 (9)	0.0728 (10)	0.0017 (8)	−0.0099 (8)	0.0323 (8)

### Geometric parameters (Å, °)

**Table:**

S1—C7	1.8238 (19)	C8A—C9A	1.501 (11)
S1—C8A	1.772 (4)	C8A—C10A	1.518 (14)
S1—C8B	1.880 (5)	C8B—C10B	1.476 (15)
O1—N1	1.205 (3)	C8B—C9B	1.529 (11)
O2—N1	1.211 (3)	C11—C12	1.495 (3)
O3A—C9A	1.226 (14)	C12—C16	1.384 (3)
O3B—C9B	1.201 (15)	C12—C13	1.370 (3)
O4—C11	1.211 (2)	C13—C14	1.374 (4)
OW1—HW1	0.813 (19)	C14—C15	1.365 (3)
OW1—HW2	0.80 (2)	C2—H2	0.9300
N1—C1	1.468 (3)	C3—H3	0.9300
N2—N3	1.386 (2)	C4—H4	0.9300
N2—C7	1.454 (2)	C5—H5	0.9300
N2—C9A	1.364 (11)	C7—H7	0.9800
N2—C9B	1.339 (10)	C8A—H8A	0.9800
N3—C11	1.360 (2)	C8B—H8B	0.9800
N4—C16	1.331 (3)	C10A—H10B	0.9600
N4—C15	1.324 (3)	C10A—H10C	0.9600
N3—HN1	0.861 (18)	C10A—H10A	0.9600
C1—C6	1.399 (3)	C10B—H10F	0.9600
C1—C2	1.383 (4)	C10B—H10E	0.9600
C2—C3	1.363 (5)	C10B—H10D	0.9600
C3—C4	1.373 (5)	C13—H13	0.9300
C4—C5	1.378 (4)	C14—H14	0.9300
C5—C6	1.390 (3)	C15—H15	0.9300
C6—C7	1.516 (2)	C16—H16	0.9300
			
S1···O2	2.999 (2)	C12···C15^ix^	3.496 (3)
S1···N1	3.466 (2)	C13···O3B^vi^	3.288 (11)
S1···S1^i^	3.5774 (7)	C13···OW1^vii^	3.286 (3)
S1···O2^i^	3.188 (2)	C14···C12^ix^	3.586 (3)
O1···O4^ii^	3.150 (4)	C14···C16^ix^	3.523 (3)
O1···C11^ii^	3.389 (3)	C14···O3A^vii^	3.420 (15)
OW1···C13^iii^	3.286 (3)	C15···C12^ix^	3.496 (3)
OW1···N4^iv^	2.803 (2)	C15···O3A^vii^	3.266 (13)
OW1···O3A^v^	2.806 (12)	C16···C14^ix^	3.523 (3)
OW1···N3^iii^	2.804 (2)	C5···HN1	3.026 (17)
OW1···O3B^v^	2.870 (12)	C9B···H5	2.9000
O2···C7	2.688 (2)	C10A···H14^vi^	3.0900
O2···S1^i^	3.188 (2)	C11···H7	2.8500
O2···N1^ii^	3.123 (3)	C12···H10E^vii^	2.9600
O2···S1	2.999 (2)	C13···HN1	2.62 (2)
O2···O2^ii^	3.177 (3)	C15···H15^x^	3.0900
O2···C1^ii^	3.348 (3)	C15···HW2^viii^	2.74 (2)
O3A···C15^iii^	3.266 (13)	C16···H10E^vii^	3.0700
O3A···C11	3.400 (16)	C16···HW2^viii^	3.04 (3)
O3A···C14^iii^	3.420 (15)	HN1···OW1^vii^	1.958 (17)
O3A···OW1^v^	2.806 (12)	HN1···C5	3.026 (17)
O3A···N3	2.720 (16)	HN1···H5	2.4100
O3B···OW1^v^	2.870 (12)	HN1···HW1^vii^	2.45 (3)
O3B···C13^vi^	3.288 (11)	HN1···HW2^vii^	2.46 (3)
O3B···N3	2.769 (15)	HN1···O3B	2.87 (3)
O4···C7	3.136 (2)	HN1···H13	2.1800
O4···C9A	3.288 (10)	HN1···C13	2.62 (2)
O4···O1^ii^	3.150 (4)	H2···O1	2.3600
O4···N2	2.689 (2)	HW1···H13^iii^	2.2900
O1···H10E^i^	2.8800	HW1···O3B^v^	2.09 (2)
O1···H8A^i^	2.9200	HW1···O3A^v^	2.02 (2)
O1···H2	2.3600	HW1···HN1^iii^	2.45 (3)
OW1···H5^iii^	2.6600	HW2···C16^iv^	3.04 (3)
OW1···H13^iii^	2.4800	HW2···N4^iv^	2.01 (2)
OW1···HN1^iii^	1.958 (17)	HW2···C15^iv^	2.74 (2)
O2···H7	2.2600	HW2···H5^iii^	2.4700
O3A···H10B	2.8900	HW2···HN1^iii^	2.46 (3)
O3A···H13^vi^	2.9000	H5···HN1	2.4100
O3A···HW1^v^	2.02 (2)	H5···HW2^vii^	2.4700
O3B···H10F	2.7000	H5···OW1^vii^	2.6600
O3B···HN1	2.87 (3)	H5···N2	2.4100
O3B···H13^vi^	2.5800	H5···C9B	2.9000
O3B···HW1^v^	2.09 (3)	H5···N3	2.7000
O4···H7	2.6300	H7···O2	2.2600
O4···H16	2.5800	H7···O4	2.6300
N1···S1	3.466 (2)	H7···C11	2.8500
N1···O2^ii^	3.123 (3)	H7···N1	2.8700
N2···O4	2.689 (2)	H8A···O1^i^	2.9200
N3···O3B	2.769 (15)	H10B···O3A	2.8900
N3···OW1^vii^	2.804 (2)	H10B···H14^vi^	2.3200
N3···O3A	2.720 (16)	H10E···C12^iii^	2.9600
N3···C5	3.167 (3)	H10E···O1^i^	2.8800
N4···OW1^viii^	2.803 (2)	H10E···C16^iii^	3.0700
N1···H7	2.8700	H10F···H14^vi^	2.4100
N2···H5	2.4100	H10F···O3B	2.7000
N3···H13	2.6500	H13···OW1^vii^	2.4800
N3···H5	2.7000	H13···N3	2.6500
N4···HW2^viii^	2.01 (2)	H13···HN1	2.1800
C1···O2^ii^	3.348 (3)	H13···HW1^vii^	2.2900
C5···C9B	3.466 (10)	H13···O3A^vi^	2.9000
C5···N3	3.167 (3)	H13···O3B^vi^	2.5800
C7···O2	2.688 (2)	H14···C10A^vi^	3.0900
C7···O4	3.136 (2)	H14···H10B^vi^	2.3200
C9A···O4	3.288 (10)	H14···H10F^vi^	2.4100
C9B···C5	3.466 (10)	H15···C15^x^	3.0900
C11···O3A	3.400 (16)	H15···H15^x^	2.4000
C11···O1^ii^	3.389 (3)	H16···O4	2.5800
C12···C14^ix^	3.586 (3)		
			
C7—S1—C8A	96.72 (17)	C13—C12—C16	117.71 (19)
C7—S1—C8B	89.56 (19)	C11—C12—C13	124.34 (17)
HW1—OW1—HW2	105 (3)	C12—C13—C14	119.5 (2)
O1—N1—C1	118.6 (2)	C13—C14—C15	118.5 (2)
O2—N1—C1	120.00 (17)	N4—C15—C14	123.6 (2)
O1—N1—O2	121.4 (2)	N4—C16—C12	123.38 (17)
N3—N2—C9A	119.9 (4)	C3—C2—H2	120.00
N3—N2—C9B	120.7 (4)	C1—C2—H2	120.00
N3—N2—C7	118.23 (15)	C2—C3—H3	120.00
C7—N2—C9B	120.9 (4)	C4—C3—H3	120.00
C7—N2—C9A	119.4 (4)	C3—C4—H4	120.00
N2—N3—C11	118.67 (16)	C5—C4—H4	120.00
C15—N4—C16	117.28 (18)	C6—C5—H5	119.00
C11—N3—HN1	124.9 (14)	C4—C5—H5	119.00
N2—N3—HN1	115.9 (14)	N2—C7—H7	109.00
N1—C1—C2	116.4 (2)	C6—C7—H7	109.00
N1—C1—C6	121.7 (2)	S1—C7—H7	109.00
C2—C1—C6	121.9 (2)	S1—C8A—H8A	109.00
C1—C2—C3	120.1 (3)	C10A—C8A—H8A	109.00
C2—C3—C4	119.8 (3)	C9A—C8A—H8A	109.00
C3—C4—C5	120.0 (3)	C9B—C8B—H8B	109.00
C4—C5—C6	122.3 (2)	C10B—C8B—H8B	109.00
C5—C6—C7	120.07 (16)	S1—C8B—H8B	109.00
C1—C6—C7	124.04 (16)	H10A—C10A—H10B	109.00
C1—C6—C5	115.89 (18)	C8A—C10A—H10C	109.00
N2—C7—C6	113.13 (13)	C8A—C10A—H10B	110.00
S1—C7—N2	103.61 (12)	H10A—C10A—H10C	109.00
S1—C7—C6	112.56 (12)	H10B—C10A—H10C	109.00
C9A—C8A—C10A	114.1 (6)	C8A—C10A—H10A	110.00
S1—C8A—C9A	106.0 (5)	C8B—C10B—H10D	109.00
S1—C8A—C10A	109.8 (5)	C8B—C10B—H10E	109.00
C9B—C8B—C10B	113.3 (7)	H10D—C10B—H10F	110.00
S1—C8B—C10B	112.3 (5)	H10D—C10B—H10E	109.00
S1—C8B—C9B	104.0 (5)	H10E—C10B—H10F	110.00
N2—C9A—C8A	113.7 (6)	C8B—C10B—H10F	109.00
O3A—C9A—C8A	124.5 (12)	C14—C13—H13	120.00
O3A—C9A—N2	121.6 (11)	C12—C13—H13	120.00
O3B—C9B—C8B	124.1 (11)	C13—C14—H14	121.00
N2—C9B—C8B	109.7 (6)	C15—C14—H14	121.00
O3B—C9B—N2	126.2 (11)	N4—C15—H15	118.00
O4—C11—N3	122.8 (2)	C14—C15—H15	118.00
N3—C11—C12	113.92 (16)	N4—C16—H16	118.00
O4—C11—C12	123.26 (16)	C12—C16—H16	118.00
C11—C12—C16	117.92 (16)		
			
C8A—S1—C7—C6	120.0 (2)	N1—C1—C2—C3	−179.5 (3)
C7—S1—C8A—C10A	−117.5 (5)	C2—C1—C6—C7	179.7 (2)
C7—S1—C8A—C9A	6.1 (5)	C1—C2—C3—C4	1.6 (5)
C8A—S1—C7—N2	−2.6 (2)	C2—C3—C4—C5	−0.2 (5)
O1—N1—C1—C6	−168.9 (3)	C3—C4—C5—C6	−1.7 (5)
O1—N1—C1—C2	9.5 (4)	C4—C5—C6—C1	2.0 (4)
O2—N1—C1—C2	−168.0 (3)	C4—C5—C6—C7	−178.2 (2)
O2—N1—C1—C6	13.7 (4)	C5—C6—C7—N2	8.9 (3)
C9A—N2—N3—C11	−86.1 (5)	C1—C6—C7—S1	71.6 (2)
N3—N2—C7—S1	−164.40 (12)	C5—C6—C7—S1	−108.16 (19)
C7—N2—N3—C11	75.9 (2)	C1—C6—C7—N2	−171.39 (19)
C9A—N2—C7—C6	−124.5 (4)	C10A—C8A—C9A—N2	112.4 (7)
C9A—N2—C7—S1	−2.3 (4)	S1—C8A—C9A—N2	−8.5 (7)
N3—N2—C7—C6	73.41 (19)	C10A—C8A—C9A—O3A	−62.0 (12)
C7—N2—C9A—C8A	7.4 (8)	S1—C8A—C9A—O3A	177.1 (9)
N3—N2—C9A—O3A	−16.3 (11)	N3—C11—C12—C13	−30.2 (3)
C7—N2—C9A—O3A	−178.0 (8)	O4—C11—C12—C13	152.0 (2)
N3—N2—C9A—C8A	169.1 (4)	O4—C11—C12—C16	−26.0 (3)
N2—N3—C11—C12	179.78 (15)	N3—C11—C12—C16	151.82 (18)
N2—N3—C11—O4	−2.4 (3)	C16—C12—C13—C14	−0.4 (3)
C16—N4—C15—C14	0.6 (3)	C11—C12—C16—N4	177.67 (18)
C15—N4—C16—C12	0.4 (3)	C11—C12—C13—C14	−178.5 (2)
N1—C1—C6—C5	177.7 (2)	C13—C12—C16—N4	−0.5 (3)
N1—C1—C6—C7	−2.1 (3)	C12—C13—C14—C15	1.3 (3)
C6—C1—C2—C3	−1.2 (5)	C13—C14—C15—N4	−1.4 (4)
C2—C1—C6—C5	−0.6 (4)		

Symmetry codes: (i) −*x*+1, −*y*+1, −*z*+1; (ii) −*x*+2, −*y*+1, −*z*+1; (iii) *x*−1, *y*, *z*; (iv) *x*−2, *y*−1, *z*; (v) −*x*+1, −*y*+1, −*z*+2; (vi) −*x*+2, −*y*+1, −*z*+2; (vii) *x*+1, *y*, *z*; (viii) *x*+2, *y*+1, *z*; (ix) −*x*+3, −*y*+2, −*z*+2; (x) −*x*+4, −*y*+2, −*z*+2.

### Hydrogen-bond geometry (Å, °)

**Table:**

*D*—H···*A*	*D*—H	H···*A*	*D*···*A*	*D*—H···*A*
N3—HN1···OW1^vii^	0.86 (2)	1.96 (2)	2.804 (2)	167 (2)
OW1—HW1···O3A^v^	0.81 (2)	2.02 (2)	2.806 (1)	163 (4)
OW1—HW2···N4^iv^	0.80 (2)	2.01 (2)	2.803 (2)	173 (3)
C5—H5···N2	0.93	2.41	2.794 (3)	104.
C7—H7···O2	0.98	2.26	2.688 (2)	105.
C13—H13···OW1^vii^	0.93	2.48	3.286 (3)	145.

Symmetry codes: (vii) *x*+1, *y*, *z*; (v) −*x*+1, −*y*+1, −*z*+2; (iv) *x*−2, *y*−1, *z*.
